# Aurora kinases are expressed in medullary thyroid carcinoma (MTC) and their inhibition suppresses *in vitro *growth and tumorigenicity of the MTC derived cell line TT

**DOI:** 10.1186/1471-2407-11-411

**Published:** 2011-09-26

**Authors:** Enke Baldini, Yannick Arlot-Bonnemains, Salvatore Sorrenti, Caterina Mian, Maria R Pelizzo, Enrico De Antoni, Silvio Palermo, Stefania Morrone, Susi Barollo, Angela Nesca, Costanzo G Moretti, Massimino D'Armiento, Salvatore Ulisse

**Affiliations:** 1 Department of Experimental Medicine, "Sapienza" University of Rome, Rome, Italy; 2 CNRS-UMR 6061 "Génétique et Développement", IFR 140 G.F.A.S., Faculté de Médecine, Université Rennes 1, Rennes, France; 3 Department of Surgical Sciences, "Sapienza" University of Rome, Rome, Italy; 4 Department of Medical and Surgical Sciences, University of Padova, Padova, Italy; 5 Veneto Institute of Oncology IOV - IRCCS, Padova, Italy; 6 Department of Internal Medicine, University of Rome Tor Vergata, Rome, Italy

## Abstract

**Background:**

The Aurora kinase family members, Aurora-A, -B and -C, are involved in the regulation of mitosis, and alterations in their expression are associated with cell malignant transformation. To date no information on the expression of these proteins in medullary thyroid carcinoma (MTC) are available. We here investigated the expression of the Aurora kinases in human MTC tissues and their potential use as therapeutic targets.

**Methods:**

The expression of the Aurora kinases in 26 MTC tissues at different TNM stages was analyzed at the mRNA level by quantitative RT-PCR. We then evaluated the effects of the Aurora kinase inhibitor MK-0457 on the MTC derived TT cell line proliferation, apoptosis, soft agar colony formation, cell cycle and ploidy.

**Results:**

The results showed the absence of correlation between tumor tissue levels of any Aurora kinase and tumor stage indicating the lack of prognostic value for these proteins. Treatment with MK-0457 inhibited TT cell proliferation in a time- and dose-dependent manner with IC_50 _= 49.8 ± 6.6 nM, as well as Aurora kinases phosphorylation of substrates relevant to the mitotic progression. Time-lapse experiments demonstrated that MK-0457-treated cells entered mitosis but were unable to complete it. Cytofluorimetric analysis confirmed that MK-0457 induced accumulation of cells with ≥ 4N DNA content without inducing apoptosis. Finally, MK-0457 prevented the capability of the TT cells to form colonies in soft agar.

**Conclusions:**

We demonstrate that Aurora kinases inhibition hampered growth and tumorigenicity of TT cells, suggesting its potential therapeutic value for MTC treatment.

## Background

Human cancer progression is associated to the acquisition by malignant cells of novel functional capabilities, which include self-sufficiency in growth signals, insensitivity to anti-growth signals, evasion of apoptosis, limitless replicative potential, sustained angiogenesis and tissue invasion and metastasis [1]. Genomic instability, an hallmark of solid tumors including the medullary thyroid carcinoma (MTC), represents the mean by which premalignant cells may acquire the above mentioned capabilities [1-4]. The increasing knowledge about the molecular processes controlling cell division has led to the identification of a number of proteins held responsible for the genetic instability. Among these are the three Aurora kinase family members, Aurora-A, -B and -C, implicated in the regulation of multiple aspects of the mitotic process including centrosome maturation and function, chromosome segregation and cytokinesis [5-9]. In particular, Aurora-A is associated with centrosomes in G2 and mitotic cells, where it regulates centrosome maturation and mitotic spindle formation. Aurora-B is localized to the chromosomes during prophase, and as chromosome condensation occurs, Aurora-B forms a complex, called chromosomal passenger complex (CPC), with INCENP (INner CENtromere Protein), survivin and borealin/dasra-B, leading to the phosphorylation of histone H3. In metaphase, the complex accumulates on the centromeres and participates to the correction of erroneous connections between cinetocores and spindle's microtubules. Successively, during the transition from anaphase to telophase, the complex dissociates from chromosomes and relocates in the spindle midzone, where Aurora-B is required for the phosphorylation of several proteins involved in spindle dynamics and contractile ring formation. Of the three kinases Aurora-C is the less known; its role appears to be similar, at least in part, to that of Aurora-B, since it exhibits analogous subcellular localization, interaction with CPC components and phosphorylation of substrates [10-12]. The expression and activity of Aurora kinases are precisely regulated during the cell cycle, since their levels are low in G1/S phase and enhanced in the G2/M phase to be decreased after mitosis. This reduction has been shown to involve the ubiquitin-proteasome pathway [9].

Alterations in Aurora kinases expression are linked to tumor progression [13-22]. The genes encoding the Aurora kinases map, in fact, into chromosomal regions that are frequently amplified in different cancer types, and overexpression of each kinase has been detected in tumor cell lines [13-22]. Moreover, it has been demonstrated that the upregulation of Aurora-A or -B causes defects in chromosome segregation and consequent aneuploidy, and induces cell malignant transformation [21-23]. In addition, tumor tissue expression of Aurora-A or Aurora-B has been shown to be a significant prognostic factor in several human malignancies, including the non-small-cell lung, breast, liver, colorectal, ovarian, and head and neck squamous cell carcinomas [24-29]. These evidences suggest an important role for Aurora kinases in cancer progression, and structure-based drug design has led to the identification of new putative drugs which efficiently inhibit Aurora kinases [16,30-32]. This may be of relevance in those cancers which do not respond well to the available antimitotic agents, including a subset of medullary thyroid cancers (MTC) [16,30]. The latter arise from the calcitonin-producing parafollicular C cells of the thyroid and accounts for about 5-8% of all thyroid cancers [33]. It develops mostly as a sporadic tumor, being hereditary in 20-30% of cases which include the familial MTC (FMTC) and the multiple endocrine neoplasia type 2 (MEN2) [34-37]. All the hereditary MTC and approximately 50% of the sporadic tumors are caused by dominant autosomal activating mutations of the RET proto-oncogene [34-37]. Over the last decades, surgery has remained the only curative therapy, and the overall survival rate of unselected patients ten years after the primary surgery is about 70%, while treatments of recurrent or persistent disease with conventional radiotherapy or chemotherapy are generally of limited value and with no benefit in terms of survival [33-35,38]. This implies that patients classification, initial surgical treatment and lack of adequate post-surgical therapy are still major problems in the management of these patients [39-41].

In the present study, we investigated the expression of the three Aurora kinases in 26 human MTC and analyzed the effects of the Aurora inhibitor MK-0457 on growth and tumorigenicity of the MTC derived cell line TT.

## Methods

### Cell line and Materials

Thyroid medullary carcinoma derived cell line TT was purchased from Interlab Cell Line Collection (Genova, Italy). Mouse monoclonal and rabbit polyclonal antibodies against β-tubulin and β-actin were from Sigma Aldrich Co. (St-Louis, MO). Rabbit polyclonal anti-Aurora-C antibody was generated against a 16 amino acid peptide of the C-terminal part of Aurora-C (aa 259-275) by Eurogentec (Seraing, Belgium). Mouse monoclonal antibodies against Aurora-A (31C1) and Aurora-B (AIM-1) were from Abcam (Cambridge, UK). The mouse monoclonal antibody anti-phospho-histone H3 (Ser10) was from Millipore (Milano, Italy). The secondary anti-rabbit and anti-mouse antibodies TRITC- and FITC-conjugated were from Jackson Laboratories (Maine, USA). The VECTASHIELD^® ^Mounting Medium with DAPI was from Vector Laboratories (Burlingame, KS). The Cell Proliferation Reagent WST-1 was acquired from Roche Diagnostics (Mannheim, Germany). The Isol-RNA lysis reagent was from Eppendorf (Milan, Italy). The Aurora kinases inhibitor MK-0457 was provided by Merck & Co. (Rahway, NJ) and Vertex Pharmaceuticals Inc. (Cambridge, MA). DNeasy Blood and Tissue kit was from Qiagen (Milan, Italy).

### Patients

The case study consists of 26 medullary thyroid cancer (MTC) patients (16 males and 10 females, mean age 52.6 yr, range 23-73 yr). All patients underwent total thyroidectomy and central neck compartment dissection. The histological diagnoses were made independently by two different histopathologists according to the World Health Organization classification [42]. Of the 26 patients 21 were assumed to have a sporadic cancer because no germline RET mutations were found, their family history was negative, and no other endocrine neoplasia was identified. The remaining 5 cases were familial MTC. Following TNM staging 5 patients were at stage I, 4 at stage II, 5 at stage III, 7 at stage IVA and 5 at stage IVC. **All the patients gave their informed consent and study approved by the local ethical committee**.

### RET analysis

All patients gave their informed consent to genetic testing. All primary MTCs were collected after surgery, immediately frozen in liquid nitrogen, and stored at -80°C. DNA was extracted from primary cancers using the DNeasy Blood and Tissue kit. RET exons 10, 11, 13, 14, 15 and 16 mutations were assessed by direct sequencing. Activating RET mutations were found in 7 (33%) of the 21 sporadic cases and in all the 5 familial cases.

### Extraction and analysis of mRNA by quantitative RT-PCR

Tissue samples were homogenized in Isol-RNA lysis reagent with the ultra-turrax, and total RNA was extracted by the acid guanidinium thiocyanate-phenol-chloroform method [43]. The purity and integrity of the RNA preparations were checked spectroscopically and by agarose gel electrophoresis before carrying out the analytical procedures. Five μg of total RNA were reverse-transcribed and the obtained cDNAs were used as template for the subsequent quantitative PCR amplifications of the Aurora-A, Aurora-B, Aurora-C and GAPDH. Controls for DNA contamination were performed omitting the reverse transcriptase during reverse transcription. Real-time PCR were performed with the LightCycler instrument (Roche Diagnostics), employing the FastStart DNA Master SYBR Green I kit. The primers used are listed in table 1. Briefly, following polymerase activation (95°C for 2 min), 40 cycles were run with 10 sec denaturation at 95°C, 10 sec annealing at 58°C and 25 sec extension at 72°C. Standard run curves were generated for each gene using five-fold dilutions of a cDNA mixture. The PCR products were visualized on 2% agarose gel, and the specificities of the different amplicons were determined by automated DNA sequencing (Primm, San Raffaele Biomedical Science Park, Milano, Italy). The calculation of data was performed with the LightCycler relative quantification software 1.0 (Roche Diagnostics).

**Table 1:** Primer sequences, exon positions and amplicon size of the different members of the Aurora kinase family.

*Gene*	*Primers*	*Exon*	*Size (bp)*
Aurora-A	Forward 5'-CTGCATTTCAGGACCTGTTAAGG-3'	1	150
	Reverse 5'-AACGCG CTGGGAAGAATTT-3'	2	
Aurora-B	Forward 5'-AACTCCTACCCCTGGCCCTA-3'	2	104
	Reverse 5'-ACAAGTGCAGATGGGGTGAC-3'	3	
Aurora-C	Forward 5'-CGCATCCTCAAGGTAGATGTG-3'	6-7	217
	Reverse 5'-GAACACACACAAAGGGAACAGAG-3'	7	
β2-Micr.	Forward 5'-TGACTTTGTCACAGCCCAAGATA-3'	2	75
	Reverse 5'-CGGCATCTTCAAACCTCCA-3'	3-4	

### Cell cultures

The medullary thyroid cancer cell line TT was established from a 77 yr old Caucasian female [44]. These cells harbours a MEN2A mutation of the RET gene (C634W) [45] and are hypodiploid with a modal chromosome number of 43 [46]. The cells have been cultured in medium Ham's F12 containing 10% FBS, 2 mM L-glutamine at 37°C in 5% CO_2 _humidified atmosphere. In all the experiments below described medium was changed every 2 days with the sole vehicle (DMSO) or fresh inhibitor (MK-0457) added.

### Proliferation assay

TT cells were cultured in 96 well plates, and treated with different concentrations of the inhibitor (5 to 1000 nM) for 6 days, or with the dose 200 nM for different periods of time (1 to 8 days). The cell proliferating reagent WST-1 was added to cells (10 μl/100 μl culture medium) 4 h before the end of the incubation period, and the cell viability was finally measured by colorimetric assay using the CM sunrise ELISA reader (Tecan Group Ltd., Switzerland).

### Flow Cytometric analysis

TT cells were cultured in absence or in presence of 200 nM MK-0457 for 6 days. Then the culture medium was collected, the cells were washed with PBS, harvested by incubation for 5 min at 37°C in PBS with 0.1% EDTA and centrifuged at 1200 rpm for 5 min together with their medium. After a wash in PBS, the cells were resuspended in 70% ice-cold ethanol, labelled with propidium iodide and analyzed for the DNA content as described [47], using the FACScalibur Flow cytometer and CellQUEST software (BD Biosciences, San Jose, CA).

### Western blot

Control and MK-0457 (200 nM for 2 days) treated cells were lysed in RIPA buffer (50 mM Tris-HCl pH 7.4, 1% NP-40, 0.5% sodium deoxycholate, 150 mM sodium chloride, 1 mM EDTA, **protease inhibitor cocktail**), sonicated and then centrifuged at 13,000 rpm for 20 min. Protein concentrations were determined by the Bradford assay. Aliquots of 30 μg of cell protein extracts were electrophoresed on a 12.5% polyacrylamide gel and transferred onto nitrocellulose membranes. The latter were then washed with TBS-T (50 mM Tris-HCl pH 7.4, 150 mM NaCl, 0.05% Tween-20), saturated with 5% low fat milk in TBS-T and then incubated at 4°C overnight with antibodies against Aurora-A (1:500), Aurora-B (1:500), Aurora-C (1:500) or β-actin (1:1000) in TBS-T. After washing, the membranes were incubated with appropriate horseradish peroxidase-conjugated secondary antibodies against mouse or rabbit IgG (1:20,000) in TBS-T and developed using the chemiluminescence Super Signal kit (Pierce).

### Colony formation in soft agar

Petri dishes of 3.5 cm diameter were first prepared by adding 3 ml of complete medium with 0.4% soft agar. TT cells cultured in standard conditions were trypsinized, centrifuged and resuspended in a single-cell suspension of 75000 viable cells/ml. The latter was mixed with complete medium containing 0.4% soft agar at a ratio 1:2 then divided in two aliquots, one of which was supplemented with 200 nM MK-0457. These suspensions were seeded onto the Petri dishes containing the solidified agar medium, 1 ml/dish, and incubated at 37°C and 5% CO_2_. Control and treated cultures were observed under microscope just after plating, to verify the absence of cell aggregates, and next periodically checked for colonies formation. After three weeks, the colonies were photographed and the acquired images were analyzed by the MetaVue software (Universal Imaging Corp., Downingtown, PA), scoring those larger than 50 μm in diameter.

### Time-lapse analysis

TT cells were cultured in absence or in presence of 200 nM MK-0457 for 24 h under a microscope Leica DM-IRBE equipped with an incubation chamber at 37°C and 5% CO_2_. Cell pictures were acquired every 5 min using the MetaVue software.

### Immunofluorescence

TT cells cultured on glass coverslips were treated or not with 200 nM MK-0457 for 6 h, then fixed in cold methanol for 5 min, washed and preincubated with 3% bovine serum albumin (BSA) in PBS for 1 h at room temperature. After three washes with PBS, the cells were incubated with the antibodies anti-Aurora-A (1:200), anti-Aurora-B (1:500), anti-Aurora-C (1:200), anti-P-histone H3 (1:2000) and/or anti-β-tubulin (1:2000) for 2 h at room temperature in PBS with 1.5% BSA. After washing, the secondary TRITC- and FITC-conjugated anti-mouse and anti-rabbit antibodies (1:200) were added in PBS with 1.5% BSA and incubated for 1 h at room temperature. The coverslips were then mounted in Vectashield mounting medium containing 1 μg/ml DAPI and observed with a microscope Leica-DMRXA. In parallel experiments cells have been cultured for 6 days in the presence or absence of the MK-0457 to assess ploidy. Cells were stained for β-tubulin and DNA, and then 100 cells for each of three different coverslips for control and MK-0457 were counted.

### Statistical analysis

The statistical significance of differences in the expression levels of the Aurora kinases and TNM stages was assessed by the analysis of variance (One way ANOVA) followed by the Tukey post ANOVA test. The results obtained following TT cell incubation in the presence or in the absence of MK-0457 were expressed as the mean ± SEM of three independent experiments. The statistical significance of data was evaluated by the Student *t*-test using the SPSS software (SPSS Inc., Chicago, Ill.). The results were considered significantly different if the pertaining p values were lower than 0.05.

## Results

### Correlation of Aurora kinases expression with tumor stage and RET mutation

**Figure 1:**
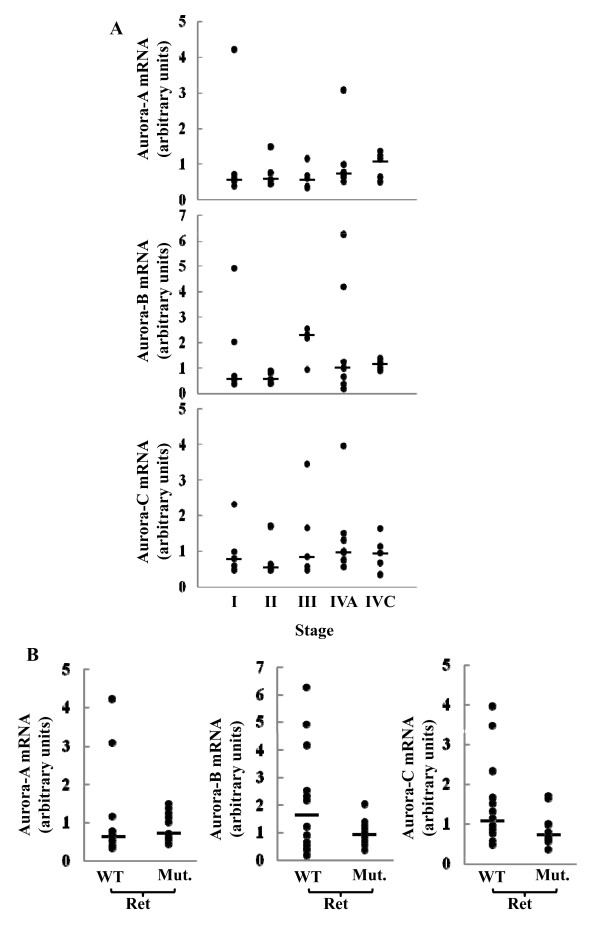
**Correlation of Aurora kinases expression with the TNM stage and RET status**. **(A) **The Aurora kinase mRNAs level in MTC tissues was quantified as described in the Materials and Methods section. The statistical analysis of differences in the expression level of the three kinases in MTC tissues at different TNM stages was assessed by the analysis of variance (ANOVA) followed by the Tukey post ANOVA test. **(B) **Aurora kinases mRNA level in MTC tissues harboring the wild type (WT) or the mutated (Mut) RET protein. The bars in the graphs indicate the median values.

To investigate the Aurora kinases expression in medullary thyroid cancer (MTC) we determined their relative mRNA tissue levels in 26 MTC and correlated them with TNM stages. As shown in figure 1 (panel A), no statistically significant variations were observed in the expression of Aurora-A, -B or -C among the different TNM stages. We then sought to verify whether the presence of activating RET mutations would affect the expression of the 3 Aurora kinases. As reported in figure 1, (panel B) no differences were found in the Aurora kinases mRNA levels between RET negative and RET positive tissues.

### Effect of MK-0457 on TT cell proliferation

**Figure 2:**
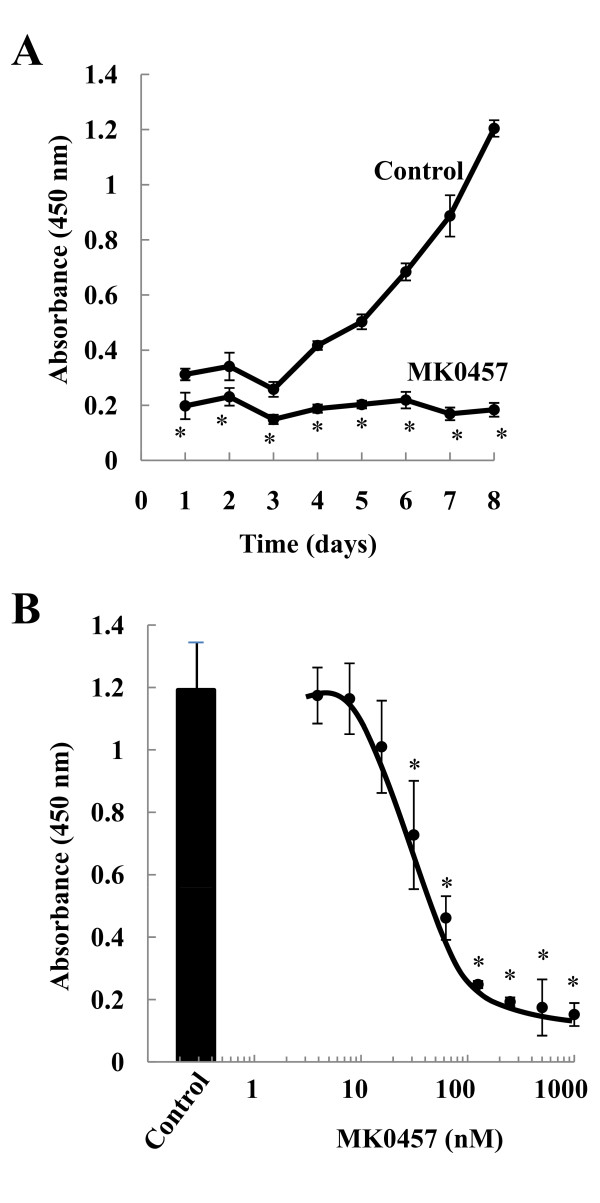
**Time- and dose-dependent effects of the MK-0457 on TT cell proliferation**. The TT cells were cultured in absence (DMSO) or in presence of 200 nM MK-0457 for different periods of time (**A**) or with different concentrations of MK-0457 (5 nM - 1000 nM) for 6 days (**B**). Data reported are representative of one out of three similar experiments. Statistical significance of data was assessed by the Student t-test. * p < 0.01.

The effect of the functional inhibition of the Aurora kinases on TT cell proliferation was evaluated on cells cultured from 1 to 8 days in presence of 200 nM MK-0457 or of the vehicle alone as control. The dose of 200 nM was used in these initial experiments since it was shown to elicit maximal response on different tumor cell types *in vitro *[30]. The results demonstrated a cytostatic effect of the MK-0457 on TT cell proliferation, which became evident as soon as 24 h (figure 2, panel A). We then evaluated the dose-dependent effects of MK-0457 on the TT cells proliferation by treating the cells for 6 days in presence of increasing concentrations of the inhibitor (5 nM to 1000 nM). The results of three independent experiments showed a dose-dependent inhibition of TT cells growth with half-maximal inhibitory concentration (IC_50_) of 49.8 ± 6.6 nM (figure 2B).

### Effect of MK-0457 on TT cell ploidy

**Figure 3:**
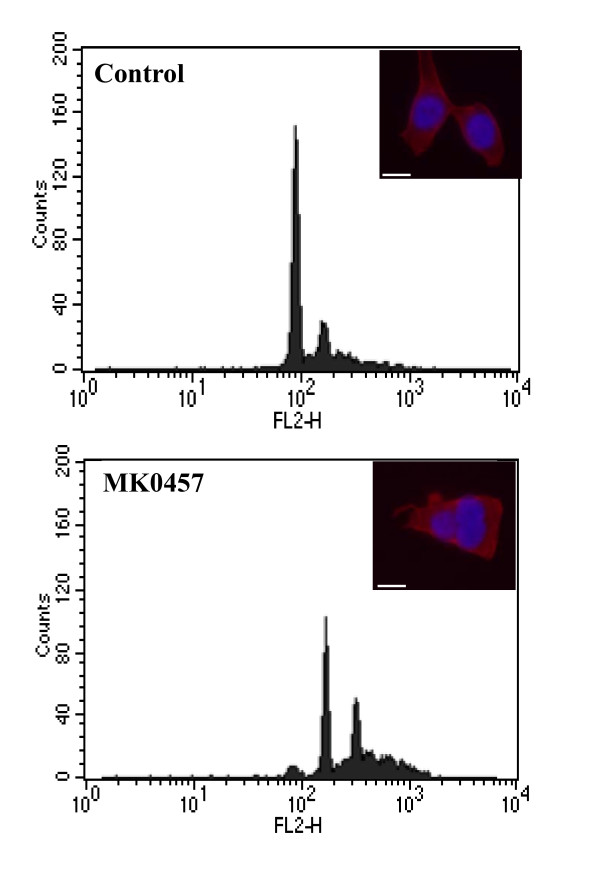
**Effects of the MK-0457 on TT cell ploidy**. Cells were incubated for 6 days with 200 nM MK-0457 or the vehicle (DMSO). At the end of the incubation time cells were fixed and analyzed by FACS. See also table 2. For the immunofluorescence experiments (insert) TT cells were exposed or not for 6 days to 200 nM MK-0457, then fixed and stained with DAPI and β-tubulin. Scale bar, 20 μm.

**Figure 4:**
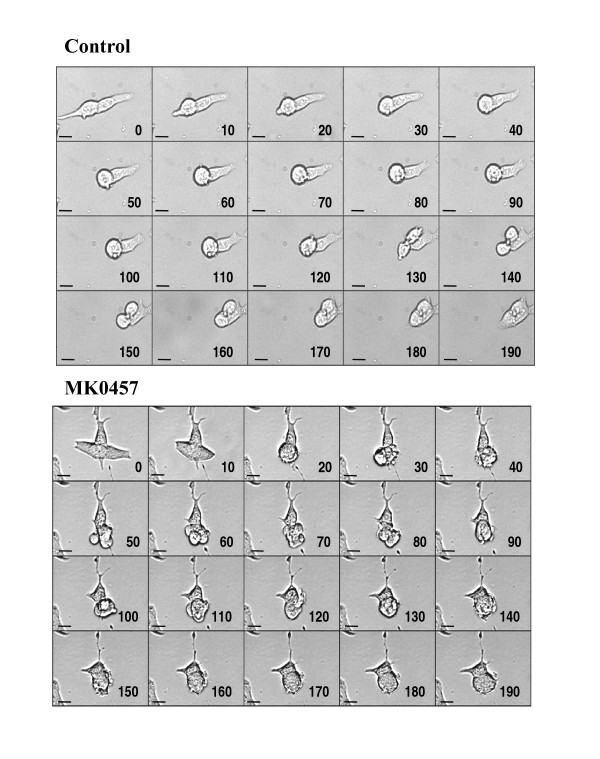
**Time-lapse analysis of control and MK-0457 treated TT cells**. Pictures of cells cultured in the absence or in the presence of 200 nM MK-0457 were recorded every 5 min during 24 h using the MetaVue software. Data reported are representative of one out of three similar experiments. Numbers in the inserts represent the minutes. Scale bar, 10 μm.

The effect of MK-0457 on TT cell cycle was evaluated by FACS analysis. Cell cultures exposed to 200 nM MK-0457 for 6 days displayed a significant reduction of cells in G0/G1 and S phases (p < 0.01) with a concomitant accumulation of cells in G2/M phase (p < 0.05). A drastic increase of polyploidy cells (p < 0.01) was also observed following MK-0457 treatment (figure 3 and table 2). On the opposite, the percentage of cells with sub-G1 nuclei was not varied. Analogous results were obtained after 3 days of treatment with the inhibitor (data not shown). These findings were confirmed by immunofluorescence experiments showing a significant (p < 0.01) increase of multinucleate cells after MK-0457 treatment, from 8.3 ± 2.4% to 67.4 ± 6.1%, (see inserts of figure 3). The time-lapse monitoring the cell cycle revealed that control cells accomplished their mitosis in about 3 h (figure 4). In contrast, MK-0457 treated cells entered mitosis but were unable to complete the cytokinesis, and finally returned to the interphasic feature (figure 4).

**Table 2:** Effects of MK-0457 on TT cell ploidy.

Cell cycle phase	Control	MK-0457	t-test
**Sub G0/G1**	0.53 ± 0.13	0.33 ± 0.12	p = 0.12
**Go/G1**	51.35 ± 4.84	3.32 ± 0.09	p < 0.01
**S**	4.94 ± 0.94	0.72 ± 0.02	p < 0.01
**G2/M**	15.03 ± 0.05	22.37 ± 2.84	p < 0.05
**Polyploid**	16.57 ± 3.25	60.59 ± 2.66	p < 0.01

### Effects of the MK-0457 on Aurora kinases expression, subcellular localization, spindle formation and histone H3 phosphorylation in TT cells

**Figure 5:**
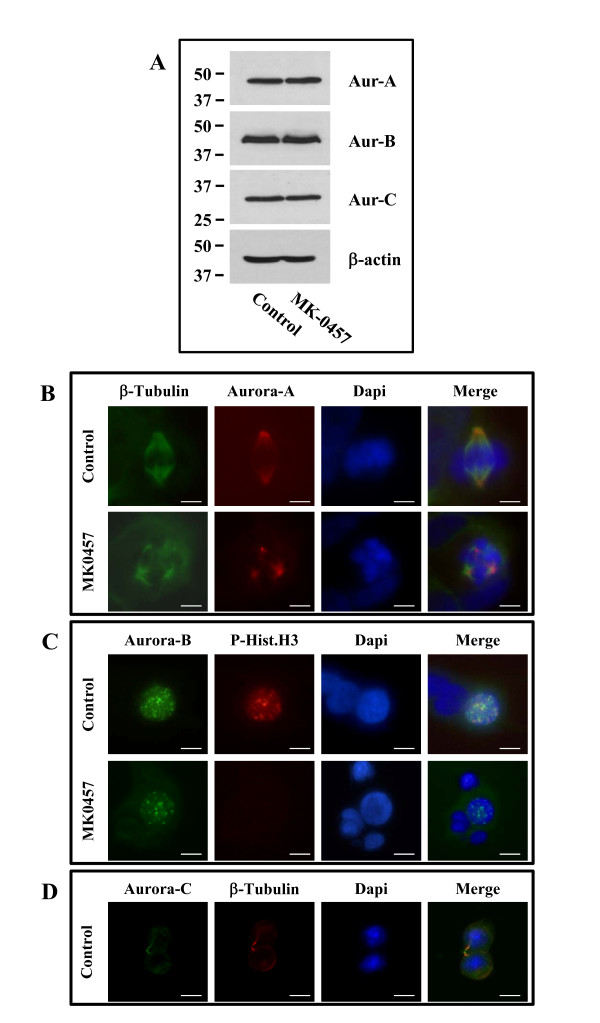
**Effects of the MK-0457 on Aurora kinases expression, subcellular localization, centrosome maturation and histone H3 phosphorylation in TT cells**. **(A) **Western blot analysis of Aurora kinases protein levels in TT cells treated or not with MK-0457 (200 nM) for 48 h. For immunofluorescence experiments TT cells were treated or not for 6 h with MK-0457 (200 nM). Cells have been stained for Aurora-A and β-tubulin **(A)**, Aurora-B and P-Histone H3 **(B) **or for Aurora-C and β-tubulin **(C)**. Scale bar, 10 μm.

We next investigated the alterations induced by MK-0457 on TT mitotic structures and proteins. To ascertain that MK-0457 effects were due to the inhibition of Aurora kinases activities and not to changes in their protein levels, we performed western blot experiments on cell protein extracts from cells treated or not with 200 nM MK-0457 for 48 h. The results showed no differences in the three protein levels between control and treated cells (figure 5, panel A). The immunofluorescence experiments showed that centrosomal localization of Aurora-A was maintained in cells exposed to MK-0457 (200 nM) for 6 h (figure 5, panel B). However, the mitotic cells had aberrant spindles characterized by shorter microtubules. In treated cells, Aurora-B localization on the condensing chromatin during prophase was also maintained, but the histone H3 phosphorylation was no longer detectable (figure 5, panel C). In control cells, Aurora-C was solely observed on the midbody of cytokinetic cells (figure 4, panel D), but following MK-0457 treatment no cells in telophase could be identified.

### Effects of MK-0457 on TT cell colony formation in soft agar

**Figure 6:**
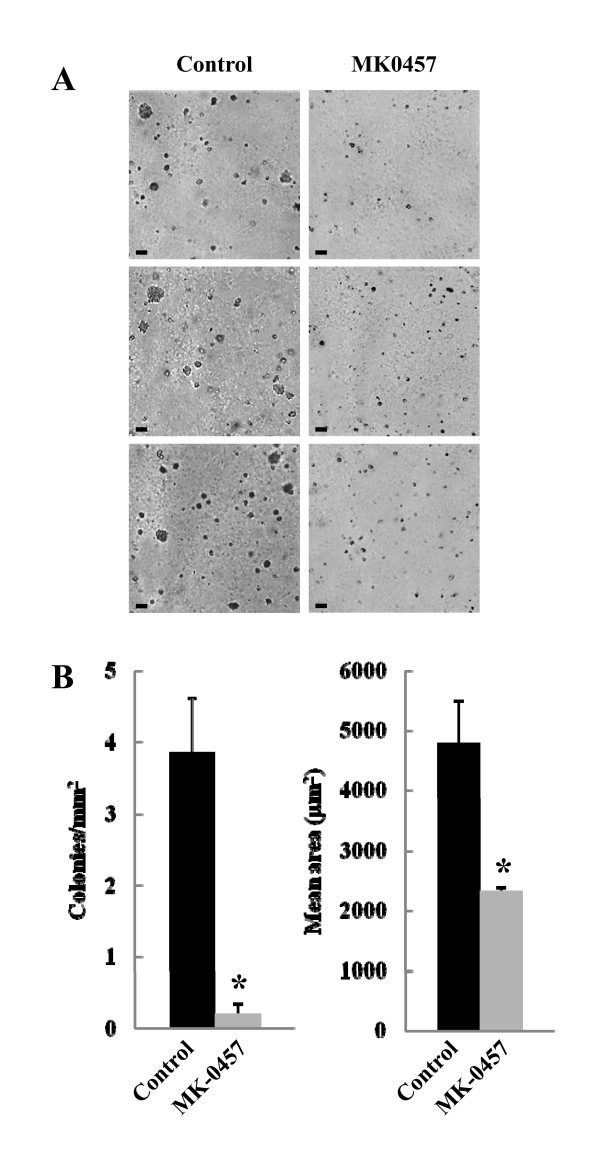
**Effects of the MK-0457 on TT cell colony formation in soft agar**. **(A) **TT cells were plated in soft agar onto 3.5 cm Petri dishes in the absence or in the presence of MK-0457 (200 nM). Treated and non-treated plates were photographed after three weeks of incubation. The colony size was determined using the MetaVue software and those larger than 50 μm in diameter were scored. Photographs reported in the figure are representative of one out of three similar experiments each performed in triplicate. Scale bar, 100 μm. (B) Effects of MK-0457 on the number and size of TT colonies in soft agar. Data reported represent the mean ± SEM of three independent experiments. *p < 0.001

We evaluated the effects of the Aurora kinases inhibitor on the ability of the TT cells to form colonies in soft agar. In these experiments the cells were cultured either in the absence or in the presence of 200 nM MK-0457 for three weeks. Control cells started to form noticeable colonies after 10 days of culture, and three weeks later 3.86 ± 0.76 colonies per mm^2^, with a mean area of 4796 ± 705 μm^2^, were scored (figure 6). Treatment with MK-0457 significantly reduced (p < 0.001) the ability of TT cells to form colonies in soft agar to 0.20 ± 0.15 colonies per mm^2^, with a mean area of 2324 ± 72 μm^2^.

## Discussion

Over the last decade, the three members of the Aurora kinase family, Aurora-A, -B and -C, involved in the regulation of multiple aspects of the mitotic process, have been identified as new oncogenes [6-8]. Their overexpression, in fact, may cause cell malignant transformation and correlates with a poor prognosis in several types of human malignancies, including lung, breast, liver, colorectal, ovarian, and head and neck squamous cell carcinomas, thus documenting their role in tumor formation and progression [13-32]. An association study, aimed to identify low-penetrance genes involved in sporadic MTC etiology, identified Aurora-A among 6 genes consistently associated with sporadic MTC risk in two-case control study [37]. However, no other information are available on the expression of the Aurora kinases in MTC. Therefore, in the present study we analyzed the possible clinical relevance of Aurora kinases in the prognosis and therapy of MTC patients. In particular, we first investigated the expression levels of all Aurora kinases in MTC tissues and attempted to correlate them with TNM stage, strongly associated with the cure and survival rates [39]. The results, obtained on a case study of 26 MTC patients, differently from what observed in other types of malignancy, indicate the absence of correlation between the expression of the three Aurora kinases and TNM stages [24-32]. Moreover, the expression level of all kinases was not varied by the presence of RET mutations, known to associate with a poor prognosis. These findings, however, remain to be corroborated on larger case studies.

Over the last few years, a number of different inhibitors of the Aurora kinases have been developed and some of them were reported to enter in Phase I clinical trials [48]. These include MK-0457, a functional pan-Aurora kinases inhibitor with inhibition constant (K_i_) ranging between 0.6 and 18 nM and showing more than 100 fold selectivity with respect to other kinases tested [30]. It inhibits tumor growth in a variety of *in vivo *xenograft models, inducing regression of leukemia, colon and pancreatic tumors at well-tolerated doses [30]. We first demonstrated that treatment of the MTC derived cell line TT with MK-0457 leads to time- and dose-dependent inhibition of proliferation, with IC_50 _of about 50 nM, in agreement with what reported on other cancer cell types [24-26].

In previous works, we and others demonstrated that Aurora-A kinase activity is required for the phosphorylation and localization of the TACC3 protein on the spindle microtubules. TACC3, in complex with the Ch-Tog protein, is essential in spindle microtubule growth and stability [14,49]; hence, alteration of TACC3 localization following MK-0457 treatment could explain, at least in part, the aberrant spindle formation in TT cells. Histone H3 is also a well recognized target of Aurora-B kinase and its phosphorylation is thought to mediate chromosome condensation during prophase [50]. In the present study, in agreement with other reports [24-26,30], we showed that MK-0457 treatment of TT cells inhibits histone H3 phosphorylation. Thus, the anti-proliferative effect of MK-0457 on TT cells is consistent with the inhibition of Aurora kinases leading to major alterations in centrosome functions, abnormal spindle formation characterized by the presence of short microtubules and mitotic arrest [24-26]. Inhibition of Aurora kinase activity has been demonstrated to generate polyploid cells as a result of multiple rounds of DNA synthesis in the absence of cytokinesis [51]. The final response to the MK-0457 is thought to be conditioned by the p53-p21-dependent post-mitotic checkpoint: cells with intact checkpoint function arrest with 4N DNA content, while those with compromised p53-dependent pathway undergo endoreduplication and apoptosis [52]. The TT cells employed in the present study possess a wild-type p53 gene and, in agreement with the above findings, we observed that MK-0457 causes abortive mitosis with accumulation of TT cells with 4N DNA content without apoptosis [53]. As a consequence, the MK-0457 treatment significantly reduces the ability of the TT cells to form colonies in soft agar [24-26]. It has to be mentioned that Merck suspended the enrollment in clinical trials of the MK-0457 due to QTc prolongation observed in one patient. However, a number of small molecule inhibitors of Aurora kinases are currently under investigation, some of which have entered clinical trials [54].

## Conclusions

In conclusion, we demonstrated that human MTC tissues express the three Aurora kinases and that their functional inhibition prevent proliferation and *in vitro *tumorogenicity of the MTC derived cells TT. These findings warrant further investigations to exploit the potential therapeutic value of Aurora kinases inhibition in the treatment of MTC patients with recurrent or persistent disease for which no effective therapies are available.

## Abbreviations

MTC: Medullary Thyroid Carcinoma; CPC: Chromosomal Passenger Complex; INCENP: INner CENtromere Protein; TNM: Tumor Node Metastasis; RET: Rearranged upon Transformation; DMSO: DiMethyl SulfOxide; PBS: Phosphate Buffered Saline; ELISA: Enzyme-Linked Immunosorbent Assay; EDTA: EthyleneDiamineTetraacetic Acid; TT: human medullary thyroid carcinoma derived cell line; IF: immunofluorescence; TACC3: transforming acidic coiled-coil 3 protein; Ch-Tog: Colonic and hepatic Tumor over-expressed protein.

## Competing interests

The authors declare that they have no competing interests.

## Authors' contributions

EB has been involved in study design, data analysis and interpretation and manuscript preparation; YAB has been involved in study design and data acquisition; SS has been involved in study design and statistical analysis of data; CM, MRP, SP, SM, SB and AN have been involved in data acquisition, analysis and interpretation and contributed to manuscript preparation and editing; CGM, MDA, EDA and SU have been involved in study concepts and design, data analysis and statistical analysis, manuscript preparation and editing. All authors read and approved the final manuscript.

## Pre-publication history

The pre-publication history for this paper can be accessed here:

http://www.biomedcentral.com/1471-2407/11/411/prepub

## References

[B1] Hanahan D Weimberg RA The Hallmark of cancer Cell 2000 100 57 70 10.1016/S0092-8674(00)81683-9 10647931

[B2] Hay ID Ryan JJ Grant CS Bergstralh EJ van Heerden JA Goellner JR Prognostic significance of nondiploid DNA determined by flow cytometry in sporadic and familial medullary thyroid carcinoma Surgery 1990 108 979 980 2247843

[B3] Bergholm U Adami HO Auer G Bergström R Bäckdahl M Grimelius L Hansson G Ljungberg O Wilander E Histological characteristics and nuclear DNA content as prognostic factors in medullary thyroid carcinoma. A nationwide study in Sweden Cancer 1989 64 135 142 10.1002/1097-0142(19890701)64:1<135::AID-CNCR2820640123>3.0.CO;2-G 2731109

[B4] Ekman ET Bergholm U Bäckdahl M Adami HO Bergström R Grimelius L Auer G Nuclear DNA content and survival in medullary thyroid carcinoma Cancer 1990 65 511 517 10.1002/1097-0142(19900201)65:3<511::AID-CNCR2820650323>3.0.CO;2-B 2297642

[B5] Ke YW Dou Z Zhang J Yao XB Function and regulation of Aurora/IpI1p kinase family in cell division Cell Res 2003 13 69 81 10.1038/sj.cr.7290152 12737516

[B6] Carmena M Earnshaw WC The cellular geography of Aurora kinases Nature Rev 2003 4 842 854 10.1038/nrm1245 14625535

[B7] Bischoff JR Plowman GD The Aurora/IpI1p kinase family: regulators of chromosome segregation and cytokinesis Trends Cell Biol 1999 9 454 459 10.1016/S0962-8924(99)01658-X 10511710

[B8] Kimura M Matsuda Y Yoshioka T Okano Y Cell cycle-dependent expression and centrosome localization of a third human Aurora/IpI1-related protein kinase, AIK3 J Biol Chem 1999 274 7334 7340 10.1074/jbc.274.11.7334 10066797

[B9] Klotzbucher A Pascreau G Prigent C Arlot-Bonnemains Y A method for analyzing the ubiquitination and degradation of Aurora-A Biol Proced Online 2002 4 62 69 10.1251/bpo35 12734567 PMC145558

[B10] Tang CJ Lin CY Tang TK Dynamic localization and functional implications of Aurora-C kinase during male mouse meiosis Dev Biol 2006 290 398 410 10.1016/j.ydbio.2005.11.036 16386730

[B11] Slattery SD Moore RV Brinkley BR Hall RM Aurora-C and Aurora-B share phosphorylation and regulation of CENP-A and Borealin during mitosis Cell Cycle 2008 7 787 795 10.4161/cc.7.6.5563 18239465

[B12] Gabillard JC Ulisse S Baldini E Sorrenti S Cremet JY Coccaro C Prigent C D'Armiento M Arlot-Bonnemains Y Aurora-C interacts with and phosphorylates the transforming acidic coiled-coil 1 protein Biochem Biophys Res Co 2011 408 647 653 10.1016/j.bbrc.2011.04.078 21531210

[B13] Ulisse S Delcros JG Baldini E Toller M Curcio F Giacomelli L Prigent C Ambesi-Impiombato FS D'Armiento M Arlot-Bonnemains Y Expression of Aurora kinases in human thyroid carcinoma cell lines and tissues Int J Cancer 2006 119 275 282 10.1002/ijc.21842 16477625

[B14] Ulisse S Delcros JG Baldini E Toller M Curcio F Giacomelli L Prigent C Ambesi-Impiombato FS D'Armiento M Arlot-Bonnemains Y Transforming Acidic Coiled-Coil 3 and Aurora-A interact in human thyrocytes and their expression is deregulated in thyroid cancer tissues Endocr Relat Cancer 2007 14 831 842 10.1677/ERC-07-0053 PMC2216418 17914111

[B15] Brinkley BR Managing the centrosome number games: from chaos to stability in cancer cell division Trends Cell Biol 2001 11 18 21 10.1016/S0962-8924(00)01872-9 11146294

[B16] Sausville EA Aurora kinases dawn as cancer drug targets Nat Med 2004 10 234 235 10.1038/nm0304-234 14991042

[B17] Dove W Aurora and the hunt for cancer-modifyng genes Nat Gen 2003 34 353 354 10.1038/ng0803-353 12923537

[B18] Bischoff JR Anderson L Zhu Y Mossie K Ng L Souza B Schryver B Flanagan P Clairvoyant F Ginther C Chan CS Novotny M Slamon DJ Plowman GD A homologue of Drosophila Aurora kinase is oncogenic and amplified in human colorectal cancer EMBO J 1998 17 3052 3065 10.1093/emboj/17.11.3052 9606188 PMC1170645

[B19] Tatsuka M Katayama H Ota T Tanaka T Odashima S Suzuki F Terada Y Multinuclearity and increased ploidy caused by overexpression of the Aurora- and IpI1-like midbody-associated protein mitotic kinase in human cancer cells Cancer Res 1998 58 4811 4816 9809983

[B20] Takahashi T Futamura M Yoshimi N Sano J Katada M Takagi Y Kimura M Yoshioka T Okano Y Saji S Centrosomal kinases, HsAIRK1 and HsAIRK3, are overexpressed in primary colorectal cancer Jpn J Cancer Res 2000 91 1007 1014 10.1111/j.1349-7006.2000.tb00878.x 11050471 PMC5926256

[B21] Miyoshi Y Iwao K Egawa C Noguchi S Association of centrosomal kinase STK15/BTAK mRNA expression with chromosomal instability in human breast cancer Int J Cancer 2001 92 370 373 10.1002/ijc.1200 11291073

[B22] Zhou H Kuang J Zhong L Kuo WL Gray JW Sahin A Brinkley BR Sen S Tumour amplified kinase STK15/BTAK induces centrosome amplification, aneuploidy and transformation Nat Genet 1998 20 189 193 10.1038/2496 9771714

[B23] Ota T Suto S Katayama H Han ZB Suzuki F Maeda M Tanino M Terada Y Tatsuka M Increased mitotic phosphorylation of histone H3 attributable to AIM-1/Aurora-B overexpression contributes to chromosome number instability Cancer Res 2002 62 5168 5177 12234980

[B24] Ogawa E Takenaka K Katakura H Adachi M Otake Y Toda Y Kotani H Manabe T Wada H Tanaka F Perimembrane Aurora-A expression is a significant prognostic factor in correlation with proliferative activity in non-small-cell lung cancer (NSCLC) Ann Surg Oncol 2007 15 547 554 18043979 10.1245/s10434-007-9653-8 PMC2244700

[B25] Reiter R Gais P Jütting U Steuer-Vogt MK Pickhard A Bink K Rauser S Lassmann S Höfler H Werner M Walch A Aurora kinase A messenger RNA overexpression is correlated with tumor progression and shortened survival in head and neck squamous cell carcinoma Clin Cancer Res 2006 12 5136 5141 10.1158/1078-0432.CCR-05-1650 16951231

[B26] Landen CN Jr Lin YG Immaneni A Deavers MT Merritt WM Spannuth WA Bodurka DC Gershenson DM Brinkley WR Sood AK Overexpression of the centrosomal protein Aurora-A kinase is associated with poor prognosis in epithelial ovarian cancer patients Clin Cancer Res 2007 13 4098 4104 10.1158/1078-0432.CCR-07-0431 17634535

[B27] Lam AKY Ong K Ho YH Aurora kinase expression in colorectal adenocarcinoma: correlations with clinicopathological features, p16 expression, and telomerase activity Hum Pathol 2008 39 599 604 10.1016/j.humpath.2007.09.001 18284933

[B28] Nadler Y Camp RL Schwartz C Rimm DL Kluger HM Kluger Y Expression of Aurora A (but not Aurora B) is predictive of survival in breast cancer Clin Cancer Res 2008 14 4455 4462 10.1158/1078-0432.CCR-07-5268 18628459 PMC5849429

[B29] Tanaka S Arii S Yasen M Mogushi K Su NT Zhao C Imoto I Eishi Y Inazawa J Miki Y Tanaka H Aurora kinase B is a predictive factor for the aggressive recurrence of hepatocellular carcinoma after curative hepatectomy Br J Surg 2008 95 611 619 10.1002/bjs.6011 18311747

[B30] Harrington EA Bebbington D Moore J Rasmussen RK Ajose-Adeogun AO Nakayama T Graham JA Demur C Hercend T Diu-Hercend A Su M Golec JM Miller KM VX-680, a potent and selective small-molecule inhibitor of the Aurora kinases, suppresses tumor growth in vivo Nat Med 2004 10 262 267 10.1038/nm1003 14981513

[B31] Arlot-Bonnemains Y Baldini E Martin B Delcros JG Toller M Curcio F Ambesi-Impiombato FS D'Armiento M Ulisse S Effects of the Aurora kinase inhibitor VX-680 on anaplastic thyroid cancer-derived cell lines Endocr Relat Cancer 2008 15 559 568 10.1677/ERC-08-0021 18430894

[B32] Ulisse S Arlot-Bonnemains Y Baldini E Morrone S Carocci S Di Luigi L D'Armiento M Inhibition of the aurora kinases suppresses in vitro NT2-D1 cell growth and tumorigenicity J Endocrinol 2010 204 135 142 10.1677/JOE-09-0257 19889886

[B33] Trimboli P Ulisse S Graziano FM Marzullo A Ruggieri M Calvanese A Piccirilli F Cavaliere R Fumarola A D'Armiento M Trend in thyroid carcinoma size, age at diagnosis and histology in a retrospective study of 500 cases diagnosed over 20 years Thyroid 2006 14 1151 1155 10.1089/thy.2006.16.1151 17123342

[B34] de Groot JW Links TP Plukker JT Lips CJ Hofstra RM RET as a diagnostic and therapeutic target in sporadic and hereditary endocrine tumors Endocr Rev 2006 27 535 560 10.1210/er.2006-0017 16849421

[B35] Schlumberger M Carlomagno F Baudin E Bidart JM Santoro M New therapeutic approaches to treat medullary thyroid carcinoma Nat Clin Pract 2008 4 22 32 10.1038/ncpendmet0717 18084343

[B36] Ball DW Medullary thyroid cancer: therapeutic targets and molecular markers Curr Opin Oncol 2007 19 18 23 10.1097/CCO.0b013e32801173ea 17133107

[B37] Ruiz-Llorente S Montero-Conde C Milne RL Moya CM Cebrián A Letón R Cascón A Mercadillo F Landa I Borrego S Pérez de Nanclares G Alvarez-Escolá C Díaz-Pérez JA Carracedo A Urioste M González-Neira A Benítez J Santisteban P Dopazo J Ponder BA Robledo M Medullary Thyroid Carcinoma Clinical Group. Association study of 69 genes in the ret pathway identifies low-penetrance loci in sporadic medullary thyroid carcinoma Cancer Res 2007 67 9561 9567 10.1158/0008-5472.CAN-07-1638 17909067

[B38] Vezzosi D Bennet A Caron P Recent advances in treatment of medullary thyroid carcinoma Ann Endocrinol 2007 68 147 153 10.1016/j.ando.2006.11.004 17391636

[B39] van Veelen W de Groot JW Acton DS Hofstra RM Höppener JW Links TP Lips CJ Medullary thyroid carcinoma and biomarkers: past, present and future J Intern Med 2009 266 126 140 10.1111/j.1365-2796.2009.02106.x 19522831

[B40] Pelizzo MR Boschin IM Bernante P Toniato A Piotto A Pagetta C Nibale O Rampin L Muzzio PC Rubello D Natural history, diagnosis, treatment and outcome of medullary thyroid cancer: 37 years experience on 157 patients Eur J Surg Oncol 2007 33 493 497 10.1016/j.ejso.2006.10.021 17125960

[B41] Roman S Lin R Sosa JA Prognosis of medullary thyroid carcinoma: demographic, clinical, and pathologic predictors of survival in 1252 cases Cancer 2006 107 2134 2142 10.1002/cncr.22244 17019736

[B42] Hedinger C Williams ED Sobin LH The WHO histological classification of thyroid tumors: a commentary on the second edition Cancer 1989 63 908 911 10.1002/1097-0142(19890301)63:5<908::AID-CNCR2820630520>3.0.CO;2-I 2914297

[B43] Chomczynsky P Sacchi P Single step method of RNA isolation by guanidinium thiocyanate-phenol-chloroform extraction Anal Biochem 1987 162 156 159 2440339 10.1006/abio.1987.9999

[B44] Leong SS Horoszewicz JS Shimaoka K Friedman M Kawinski E Song MJ Zeigel R Chu TM Baylin SB Mirand EA Andreoli M, Monaco F, Robbins J A new cell line for study of human medullary thyroid carcinoma Advances in thyroid neoplasia 1981 Rome, Field Educational Italia 95 108

[B45] Carlomagno F Salvatore D Santoro M de Franciscis V Quadro L Panariello L Colantuoni V Fusco A Point mutation of the RET proto-oncogene in the TT human medullary thyroid carcinoma cell line Biochem Biophys Res Co 1995 207 1022 1028 10.1006/bbrc.1995.1287 7864888

[B46] Marsh DJ Theodosopoulos G Martin-Schulte K Richardson AL Philips J Röher HD Delbridge L Robinson BG Genome-wide copy number imbalances identified in familial and sporadic medullary thyroid carcinoma J Clin Endocrinol Metab 2003 88 1866 1872 10.1210/jc.2002-021155 12679485

[B47] Chang D Chen F Zhang F McKay BC Ljungman M Dose-dependent effects of DNA-damaging agents on p53-mediated cell cycle arrest Cell Growth Differ 1999 10 155 162 10099829

[B48] Boss DS Beijnen JH Schellens JHM Clinical experience with Aurora kinase inhibitors: a review Oncologist 2009 14 780 793 10.1634/theoncologist.2009-0019 19684075

[B49] Mori D Yano Y Toyo-oka K Yoshida N Yamada M Maramatsu M Zhang D Saya H Toyoshima YY Kinoshita K Wynshaw-Boris A Hirotsune S NDEL1 phosphorylation by Aurora-A kinase is essential for centrosomal maturation, separation and TACC3 recruitment Mol Cell Biol 2007 27 352 367 10.1128/MCB.00878-06 17060449 PMC1800650

[B50] Crosio C Fimia GM Loury R Kimura M Okano M Zhou M Sen S Allis CD Sassone-Corsi P Mitotic phosphorylation of histone H3: spatio-temporal regulation by mammalian aurora kinases Mol Cell Biol 2002 22 874 885 10.1128/MCB.22.3.874-885.2002 11784863 PMC133550

[B51] Kawasaki A Matsumura I Miyagawa Ji Ezoe S Tanaka H Terada Y Tatsuka M Machii T Miyazaki H Furukawa Y Kanakura Y Downregulation of an AIM-1 kinase couples with megakaryocytic polyploidization of human hematopoietic cells J Cell Biol 2001 22 275 287 10.1083/jcb.152.2.275 PMC2199624 11266445

[B52] Gizatullin F Yao Y Kung V Harding MW Loda M Shapiro GI The Aurora kinase inhibitor VX-680 induces endoreduplication and apoptosis preferentially in cells with compromised p53-dependent postmitotic checkpoint function Cancer Res 2006 66 7668 7677 10.1158/0008-5472.CAN-05-3353 16885368

[B53] Yoshimoto K Iwahana H Fukuda A Sano T Saito S Itakura M Role of p53 mutations in endocrine tumorigenesis: mutation detection by polymerase chain reaction-single strand conformation polymorphism Cancer Res 1992 52 5061 5064 1516062

[B54] Green MR Woolery JE Mahadevan D Update on Aurora kinase targeted therapeutics in oncology Expert Opin Drug Discov 2011 6 291 307 10.1517/17460441.2011.555395 21556291 PMC3088914

